# Small but Select

**Published:** 1885-11

**Authors:** 


### SMALL BUT SELECT.

The *New York Medical Journal*, not liking the play house as it has been built by the other children, proposes to go off to another corner and build one of its own. It proposes a new "National Association," and offers a specific plan upon which to build it. The plan consists in having it so small, so select, and "so honorable, that membership in it would carry the highest reward that medical men here [below?] would have to hope for." It also provides that a convention be called of eminent men from all parts of the country [i. e., from Philadelphia and New York—ED.], and that this convention appoint one-half of the members of the proposed association—the remaining half to be filled up later."

That is to say, the new concern is to be composed of the cream of the American Medical Profession as skimmed by Hays, Shrady et al.—in other words, of the bolters. The plan is, that the convention of eminent men from all parts of Philadelphia and New York City are to appoint half the members—that is to say, *themselves*—and "the other half to be filled up later;"—that is to say, by their friends; a real nice little admiration - Narcissus-by - the-stream-mutual - tickle-me-and - I'll-tickle you tea party, whose chief creed, we suggest, shall read thus: We thank Thee, O Lord, that we are not as other men are—being considerably more eminent; likewise more scientific, and *also* more honorable.

